# Effect of Energy Restriction on Eating Behavior Traits and Psychobehavioral Factors in the Low Satiety Phenotype

**DOI:** 10.3390/nu11020245

**Published:** 2019-01-22

**Authors:** Vicky Drapeau, Raphaëlle Jacob, Shirin Panahi, Angelo Tremblay

**Affiliations:** 1 Faculty of Educational Sciences, Department of Physical Education, Laval University, Quebec, QC G1V OA6, Canada; shirin.panahi.1@ulaval.ca; 2 Quebec Heart and Lung Institute Research Center, Quebec, QC G1V 4G5, Canada; raphaelle.jacob.1@ulaval.ca (R.J.); angelo.tremblay@kin.ulaval.ca (A.T.); 3 Institute of Nutrition and Functional Foods (INAF), Quebec, QC G1V OA6, Canada; 4 School of Nutrition, Laval University, Quebec, QC G1V OA6, Canada; 5 Faculty of Medicine, Department of Kinesiology, Laval University, Quebec, QC G1V OA6, Canada

**Keywords:** energy restriction, eating behaviors, psychobehavioral factors, satiety responsiveness

## Abstract

Studies have shown that individuals with low satiety efficiency may be more susceptible to weight gain, but little is known about the effect of weight loss intervention outcomes in these individuals. This study aimed to evaluate the impact of an energy-restricted weight loss intervention on eating behavior traits and psychobehavioral factors in individuals differing in their satiety responsiveness. A pooled cohort of individuals who were overweight or obese (*n* = 100; aged 39 ± 9 years) participating in a 12- to 15-week weight loss program targeting an energy deficit of 500–700 kcal/day were included in this study. Satiety responsiveness was determined by a median split of the mean satiety quotient based on appetite sensations measured in response to a test meal at baseline (low satiety responsiveness (LSR) vs. high satiety responsiveness (HSR)). Anthropometric variables, eating behavior traits, psychobehavioral factors, and ad libitum energy intake were assessed before and after the intervention. Although similar weight loss was observed between the LSR and HSR groups (−3.5 ± 3.2 vs. −3.8 ± 2.8 kg, *p* = 0.64) in response to an energy-restricted weight loss intervention, changes in eating behavior traits were different between groups. Individuals with LSR had a higher increase in cognitive restraint (+5.5 ± 4.1 vs. +3.5 ± 3.5, *p* = 0.02) and some of its subscales and a lower decrease in situational susceptibility to disinhibition (−0.6 ± 1.1 vs. −1.2 ± 1.3, *p* = 0.02) in response to the intervention compared to the HSR group. In conclusion, energy-restricted weight loss intervention seems to trigger undesirable changes in some eating behavior traits in individuals more vulnerable to overeating, which could increase their susceptibility to weight regain.

## 1. Introduction

Although modern obesogenic environments have led to a marked increase in body weight over the past few decades [1], a large variability in weight gain among the population has been observed. Individual, environmental, biological, and genetic differences may explain this variability [2,3]; however, there may be specific factors that cause certain individuals to eat beyond their energy needs and increase their susceptibility for weight gain. 

Differences in appetite control have been suggested to play an important role in energy balance, eating behavior traits, and body weight [2,4,5]. Clinical studies have observed that some individuals reported a poor relationship between their food intake and appetite sensations (e.g., hunger and fullness), suggesting that they experience a weakened satiety efficiency [6]. This weakened postprandial inhibitory response was also highlighted in a group of individuals with obesity and who were characterized by impaired satiety signals in response to a test meal compared to a control group of individuals who had a normal weight or obesity [5]. Although this weak satiety responsiveness, also identified as the “low satiety phenotype”, has been primarily observed in individuals with obesity, it has also been observed in those with normal body weight [6,7]. This altered satiety response in some individuals has important clinical implications given that differences in satiety signaling may be involved in overeating and susceptibility to weight gain [8].

In research settings, the satiety quotient (SQ), a marker of satiety responsiveness, has been used to characterize the low satiety phenotype and classify individuals according to their satiety signaling responsiveness in response to a standardized meal (i.e., individuals with low satiety responsiveness (LSR) or high satiety responsiveness (HSR)). Our previous work has shown that individuals characterized by a low SQ had higher ad libitum energy intake (as well as relative energy intake, i.e., energy intake beyond energy needs) under both experimental and free-living conditions [7], suggesting that adults with weaker satiety responsiveness may be more vulnerable to overeating, and thus, to weight gain.

Furthermore, some studies have identified distinct psychobehavioral and biological characteristics also indicating that individuals with LSR are at a higher risk of overeating and weight gain. Accordingly, individuals characterized by a low SQ in response to a fixed meal have been associated with a greater implicit wanting for high-fat foods, a higher level of disinhibition, a lower feeling of control over food cravings [9], a tendency to have higher levels of susceptibility for hunger triggered by external cues and night eating symptoms [10], which are all behavior traits that have been related to overeating, obesity, and weight gain [11,12,13]. LSR has also been associated with a tendency for greater anxiety and blunted cortisol response after a meal, suggesting a dysregulation with the hypothalamic-pituitary-adrenal (HPA) axis [10], a condition that has been associated with obesity [14]. 

To our knowledge, very few studies have explored how individuals with this phenotype respond to a weight loss intervention. In one previous study, no relationship between satiety responsiveness and weight loss was found [15]. However, in a more recent study, a greater decrease in body weight and body mass index (BMI) was observed in individuals with high satiety responsiveness (HSR) compared to those with LSR, irrespective of the intervention [16]. Since weight loss is often associated with non-optimal changes in appetite sensations [17] and eating and psychobehavioral traits, such as an increase in cognitive restraint [18,19,20] and even depressive symptoms [18,20], it can be postulated that individuals characterized by LSR may be more resistant to weight loss and experience less optimal changes in their eating and psychobehavioral profiles when submitted to a weight loss intervention based on energy restriction. Therefore, the objective of this study was to compare the impact of an energy-restricted weight loss program on body weight loss, eating behavior traits, and psychobehavioral factors between individuals with low and high satiety responsiveness.

## 2. Materials and Methods 

### 2.1. Participants 

The participants in this study were from a pooled cohort of individuals in the control groups of three weight loss interventions conducted at Laval University (i.e., participants who received a placebo and the same diet-based weight-reducing intervention) [21,22,23]. Inclusion criteria were: ages 20 to 55 years, overweight or obese, apparent good health, no medications that would affect study outcomes, sedentary to moderately active (i.e., low-intensity physical activities, such as brisk walking, three times/week or less, not more than 30 min/session), consumption of less than five cups of coffee/day, consumption of two or less alcohol drinks/day or less than ten alcohol drinks/week, body weight variation of less than ±4 kg for at least two months prior to the study, and premenopausal status for women. Written informed consent was provided by each participant and all studies were approved by the Laval University Ethics Committee. 

### 2.2. Diet-Based Weight-Reducing Intervention

All participants underwent the same diet-based weight loss program, which included a dietary plan and supervision by a trained registered dietitian every two weeks. The dietary intervention was based on an energy restriction of 500 to 700 kcal/day (2090–2926 kJ/day) for 12–15 weeks. The dietary plan was based on the use of a food exchange system for individuals with diabetes from the association Diabète Québec, which recommends portions of healthy foods to be consumed in each food category [24]. Participants were evaluated before (pre) and after (post) the weight loss program. A summary of the description of the three initial studies is provided in Table 1.

### 2.3. Anthropometric Measurements 

Body weight was measured to the nearest 0.1 kg using a digital scale, and height to the nearest 0.1 cm using a standard stadiometer. BMI was calculated as body weight divided by height squared (kg/m^2^). Waist circumference was measured at the line between the bottom of the last rib and top of the iliac crest. These anthropometric measurements were performed according to standardized procedures recommended by The Airlie Conference [25].

### 2.4. Subjective Appetite Sensations and Satiety Responsiveness

Appetite sensations were assessed in response to a standardized breakfast in order to determine participant satiety responsiveness. Participants consumed a standardized breakfast test meal consisting of white bread, butter, peanut butter, cheddar cheese, and orange juice within a 20 min period. The meal was designed to have 14, 42, and 44% of total energy as protein, fat, and carbohydrates, respectively, and an energy content of 733 kcal for men and 599 kcal for women. Appetite sensations were assessed by 150 mm Visual Analogue Scales (VAS) adapted from Hill and Blundell [26]. The following questions were asked: (1) Desire to eat: How strong is your desire to eat? (very weak–very strong); (2) Hunger: How hungry do you feel? (not hungry at all–as hungry as I have ever felt); (3) Fullness: How full do you feel? (not full at all–very full); and (4) Prospective food consumption (PFC): How much food do you think you could eat? (nothing at all–a large amount). VAS measurements were always performed in the same environment (i.e., alone, at the same table, same room with the same lighting and free of potentially confounding factors (i.e., odours, sounds, visual stimuli, individuals in the room, etc.). The satiety responsiveness of each individual was determined based on the mean SQ calculated for each appetite sensation using the following equation adapted from Green et al. [27]:

$$\mathrm{SQ}=\frac{\mathrm{fasting}\;\mathrm{appetite}\;\mathrm{sensations}\;\left(\mathrm{mm}\right)-\mathrm{mean}\;\mathrm{of}\;\mathrm{the}\;60\;\min\;\mathrm{post}-\mathrm{meal}\;\mathrm{appetite}\;\mathrm{sensations}\;\left(\mathrm{mm}\right)}{\mathrm{Energy}\;\mathrm{content}\;\mathrm{of}\;\mathrm{the}\;\mathrm{test}\;\mathrm{meal}\;\left(\mathrm{kcal}\right)}\times 100$$

The reproducibility of this SQ adaptation has been validated by our team over two to four weeks [10]. The SQ was used to characterize participants as either having a low or high satiety responsiveness. A higher SQ represents stronger appetite responses to the ingested food whereas a lower SQ represents a weaker response.

### 2.5. Ad Libitum Energy Intake

The buffet-type meal was composed of a variety of cold foods which varied in macronutrient composition to measure ad libitum energy intake according to procedures previously described [28]. Participants were instructed to eat until they were “comfortably full” over 30 min. Participants’ food preferences were previously verified with a questionnaire using a scale from 0 to 5 (0: don’t like at all, to 5: like very much). If they rated more than 50% of the foods as lower than 3, they were not eligible for this study. The ad libitum buffet-type test meal was provided in the laboratory under the same conditions as the standardized breakfast test meal. All foods were weighed to the nearest 0.1 g immediately before and after the test meal. Computer software (Nutrific, Laval University, Québec, QC, Canada) linked to the Canadian Nutrient File (versions 1997 and 2005) was used to enter the amount of food eaten in order to determine the ad libitum energy intake as well as the macronutrient composition of the ingested food [29].

### 2.6. Eating Behavior Traits

The Three-Factor Eating Questionnaire (TFEQ) was completed to measure the three main dimensions of human eating behavior traits: cognitive restraint, disinhibition, and susceptibility to hunger. This questionnaire includes a total of 51 items where 36 items are in a true or false format, whereas the remaining items required participants to select from a choice of four responses that varied in the level of agreement with a particular statement. Responses were scored 0 or 1 and summed, with higher scores denoting higher levels of eating disturbances. This questionnaire has been shown to have good reliability and validity [30]. The TFEQ also permits the identification of more specific eating behavior traits, such as flexible and rigid control [30], strategic dieting behavior, attitude to self-regulation, avoidance of fattening foods as TFEQ-restraint subscales, habitual susceptibility, emotional susceptibility, and situational susceptibility as TFEQ-disinhibition subscales and internal and external locus for hunger as TFEQ-Hunger subscales [31]. The Binge Scale (BES) [32] was used to assess binge-eating tendencies. It is a 16-item self-reported questionnaire where higher scores are positively correlated with higher levels of binge eating. The State-Trait Food Cravings Questionnaire (FCQ) includes 15 and 39 items, respectively, which measure state and trait dimensions of food cravings (i.e., the intense desire to consume a particular food or food type that is difficult to resist in general (Trait) or at a specific moment (State)) [33]. The FCQ-State measures the desire to eat, anticipation of positive reinforcement or relief from negative states, lack of control over eating, and craving as a physiological state, while the FCQ-Trait measures more stable dimensions of cravings, including intention to eat, positive reinforcement, negative reinforcement, lack of control, preoccupation with food, feelings of hunger, negative effect, cue-dependent eating, and guilty feelings. A higher FCQ score represents a higher susceptibility for a specific food craving.

### 2.7. Psychobehavioral Factors

The Beck Depression Inventory (BDI) [34] was used to assess depressive symptoms. The BDI has 21 questions with a higher score denoting higher depressive symptoms. The State-Trait Anxiety Inventory (STAI) [35] was used to measure anxiety symptoms at a specific moment (State) or in general (Trait). The STAI scales have 40 items and higher scores on the scales indicate higher levels of anxiety. Finally, the Pittsburgh Sleep Quality Index (PSQI) [36] was completed to determine sleep duration (self-reported item) and sleep quality (total score) over the last month. A higher global score on this questionnaire indicates poor sleep quality. These questionnaires were selected on the basis of each behavior’s influence on appetite sensations and eating behavior traits.

### 2.8. Procedures

On the first visit, participants attended the laboratory after a 12 h overnight fast. They were then instructed to eat a standardized breakfast and complete the VAS before, immediately after, and 10, 20, 30, 40, 50, and 60 mins after breakfast. During the period between the standardized breakfast test meal and buffet-type meal, participants were instructed to complete the questionnaires. After the standardized breakfast test meal, each participant relaxed in a quiet room and was instructed not to eat or drink anything, except water, until lunch time. After the breakfast test meal (i.e., 3.5 h later), each participant was provided with the buffet-type test meal to measure ad libitum energy intake. This 5 h test period was held before (pre) and after (post) the diet-based weight loss intervention. 

### 2.9. Statistical Analysis

Participants were divided in two satiety responsiveness groups using the SQ median (10.1 mm/100 kcal), (i.e., LSR group identified as individuals with a mean SQ < 10.1 mm/100 kcal and HSR group identified as individuals with a mean SQ ≥ 10.1 mm/100 kcal). This cut-off was based on our previous studies [10,15]. Group differences in baseline sex and age were assessed using the Chi-Square or general linear model (GLM) and group differences in baseline anthropometric variables, SQ and fasting appetite sensations were assessed using GLM adjusted for sex due to a sex difference between groups. Group differences in baseline eating behavior traits and psychobehavioral factors were assessed using GLM adjusted for sex and baseline BMI. Pearson’s correlations adjusted for sex and baseline BMI were conducted to assess the associations between baseline eating behavior traits, psychobehavioral factors, and SQ. Mixed linear models for repeated measures with Tukey-Kramer’s post hoc test were conducted to assess the changes in body weight, eating behavior traits, psychobehavioral factors, SQ, and ad libitum energy intake between groups over time. The effect of time, group, and their interaction were treated as fixed effects, and within-subject correlation was considered in all models. All mixed linear models were adjusted for sex, baseline BMI, duration of the weight loss intervention, and prescribed energy restriction (i.e., condition A: −700 kcal/day for 15 weeks and condition B: −500 kcal/day for 12 weeks). General linear models adjusted for sex and BMI at the related measurement time-point were also used to assess group differences in energy intake at the buffet before and after the intervention. This latter model was also adjusted for weight loss duration and prescribed energy restriction. Data are reported as means ± standard deviations (SD). The skewness and the normality of the model residuals were considered, and data were log-transformed when required. Statistical significance was considered at *p* < 0.05. All statistical analyses were performed using SAS software, version 9.4 (SAS Institute, Cary, NC, USA). 

## 3. Results

### 3.1. Baseline Participant Characteristics 

A total of 100 individuals (29 men; 71 women) participated in this study. After adjustment for sex, no significant differences were observed between LSR and HSR for body weight, height, BMI, and waist circumference (Table 2). As expected, the SQ (mean and for each appetite sensation) was significantly lower in the LSR group compared to the HSR group. Fasting hunger, desire to eat, and PFC were significantly lower and fasting fullness significantly higher in the LSR group compared to the HSR group. 

### 3.2. Baseline Eating Behavior Traits and Psychobehavioral Factors 

Before the intervention, the LSR group expressed a higher level of external locus for hunger as well as a higher PSQI total score, indicating lower sleep quality compared to the HSR group (Table 3). Moreover, the LSR group also had a higher present-state anxiety score before the intervention compared to the HSR group. Accordingly, present-state anxiety was also negatively associated with SQ (*r* = −0.38, *p* = 0.008). No other correlations at baseline were statistically significant (data not shown).

### 3.3. Changes in Body Weight, Satiety Efficiency, Eating Behavior Traits, and Psychobehavioral Factors

Similar weight loss was observed between the LSR and HRS groups (−3.5 ± 3.2 vs. −3.8 ± 2.8 kg, respectively, time effect *p* < 0.0001, group × time effect *p* = 0.64) in response to the prescribed energy restriction. Changes in satiety efficiency in response to the intervention was also similar between LSR and HSR groups (LSR pre 6.0 ± 2.6 vs. post 8.0 ± 5.4; HSR group pre 14.8 ± 3.5 vs. post 15.2 ± 4.4; time effect *p* = 0.009; group × time effect *p* = 0.07).

**Figure 1:**
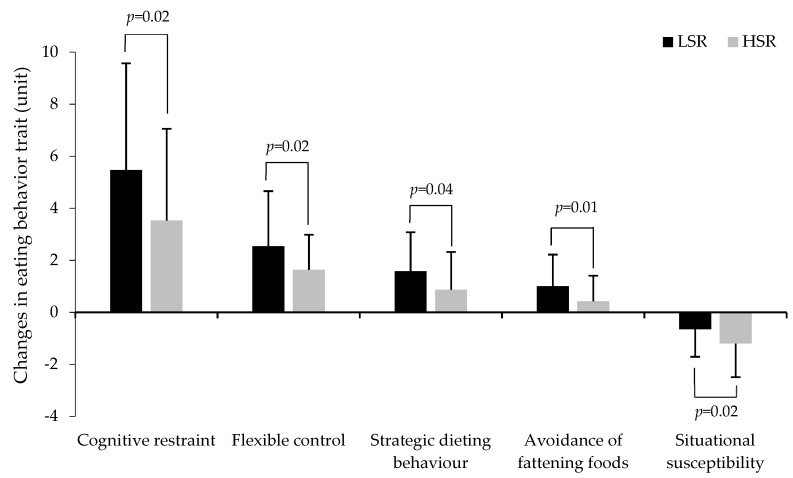
Changes in eating behavior traits in response to the weight loss intervention in the LSR group compared to the HSR group. LSR: Low satiety responsiveness, HSR: high satiety responsiveness (*n* = 88 to 98). *p* values indicate a group by time interaction for changes in eating behavior traits in response to the intervention.

After adjusting for sex and baseline BMI, a group by time interaction was observed for cognitive restraint, flexible control, strategic dieting behavior, avoidance of fattening foods, and situational susceptibility to disinhibition (Table 3). Accordingly, except for avoidance of fattening foods which increased only in LSR, both groups showed a significant increase in cognitive restraint and its subscales, flexible control, and strategic dieting behavior. However, the increase in cognitive restraint, flexible control, and strategic dieting behavior in response to the intervention was significantly higher in the LSR group than in the HRS group (Figure 1). Both groups also showed a significant decrease in situational susceptibility to disinhibition, but the decrease was significantly lower in the LSR group compared to the HSR group (Figure 1). Additional adjustments for baseline corresponding behavioral values did not significantly change the main overall findings (data not shown).

### 3.4. Ad Libitum Energy Intake at the Buffet-Type Meal 

**Figure 2:**
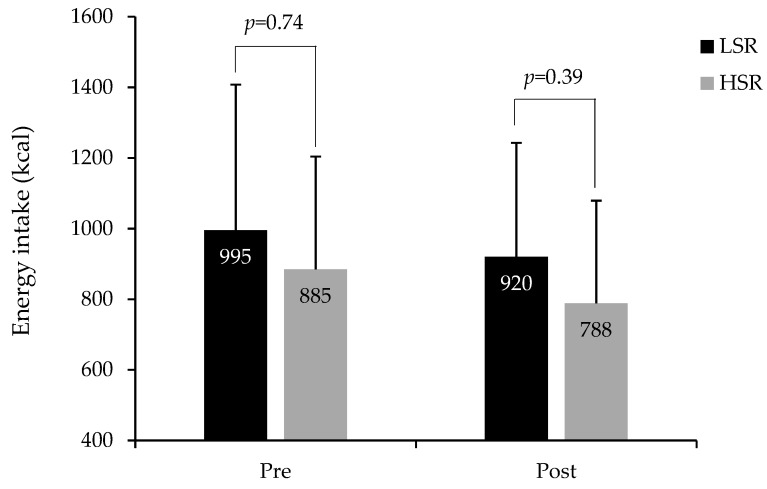
Measured energy intake at the ad libitum buffet-type meal in LSR and HSR groups at baseline (pre) and after the weight loss intervention (post). LSR: Low satiety responsiveness (pre *n* = 50; post *n* = 49), HSR: high satiety responsiveness (pre *n* = 49; post *n* = 49) (total *n* = 98 to 99). *p* values indicate group differences in energy intake as per general linear models (GLM) adjusted for sex and BMI at the related measurement time point. GLM for group difference in post-intervention energy intake was further adjusted for weight loss duration and prescribed energy restriction.

Compared to baseline values, ad libitum energy intake was lower after the intervention in both groups (time effect *p* = 0.007). Even though no significant group by time interaction was observed for energy intake in response to the intervention (*p* = 0.78), energy intake at the post-intervention buffet-type meal was 132 kcal higher in the LSR compared to the HRS group (Figure 2). This difference was, however, not significant after adjusting for weight loss duration and prescribed energy restriction, sex and post-intervention BMI (*p* = 0.39).

## 4. Discussion

The literature shows large individual variability in susceptibility to weight gain and obesity as well in weight loss and/or weight regain in response to a diet-based intervention. It has been proposed that differences in baseline satiety responsiveness may explain these variations. Although similar weight loss was observed between individuals with low and high satiety responsiveness, the energy restriction was associated with less optimal changes in some, but not all, eating behavior traits in individuals expressing LSR compared to those with HSR. These results suggest that individuals with an altered satiety responsiveness, i.e., low satiety phenotype, are able to lose body weight under energy restriction, but may be at higher risk of weight regain following an energy-restricted diet.

This study confirmed that, among individuals who were overweight or obese, differences in appetite control exist, and the LSR (i.e., lower SQ) is also associated with behavioral traits associated with higher susceptibility to overeating. Accordingly, individuals with low satiety responsiveness were more susceptible to eat in response to hunger triggered by external food cues, such as the smell of food or seeing food. They also reported poor sleep quality and a tendency to have shorter sleep duration, and reported higher present-state anxiety. Most of these behavioral traits have been associated with impaired appetite control and/or weight gain [11,13,37,38,39]. These results are concordant with our previous studies, but also with other studies showing that individuals with LSR have higher measured and reported energy intake [7,15], higher levels of disinhibition, lower control over food cravings, greater wanting for high-fat foods [9], shorter sleep duration [40], blunted cortisol response to a meal and a tendency for higher levels of anxiety and night-eating symptoms [10]. Moreover, this higher susceptibility to overeating was also concordant with what was observed at the buffet-type meal after the intervention, where individuals with LSR ate spontaneously more than those with HSR; yet, this was not statistically significant after adjusting for sex and BMI at this time-point. Taken together, these results suggest that individuals characterized by a low satiety efficiency are more at risk for overeating, especially in the presence of food cues. 

Although individuals with low LSR had a behavioral profile that increases their risk of overeating, weight loss resistance was not observed in these individuals when submitted to an energy-restricted diet. This result does not support our hypothesis, but is concordant with a previous weight loss study based on energy restriction [15]. Interestingly, these results are in contrast with one of our recent studies which investigated the impact of a weight loss intervention based on a highly satiating diet without a specific energy restriction in individuals with LSR or HSR [16]. Accordingly, a lower decrease in body weight was observed in individuals with LSR compared to those with HSR irrespective of the intervention groups (i.e., control or satiating diet) [16]. However, a significant decrease in fat mass percentage was observed in response to the satiating diet compared to control, yet this effect was similar in both satiety responsiveness groups [16]. These different results could be explained by the fact that the weight loss intervention in the highly satiating diet study was not based on energy restriction, but only on guidelines promoting a satiating diet. Moreover, in this study, even though individuals in the LSR group had less body weight loss in response to the intervention, their satiety responsiveness (i.e., SQ) was markedly improved in the satiating diet condition compared to the HSR group [16]. In the present study, similar changes in satiety responsiveness were observed in both HSR and LSR individuals in response to the diet intervention; yet the latter group was still considered “LSR” after weight loss. Collectively, these results suggest that the satiety responsiveness does not influence weight loss when the intervention is based on energy restriction compared to a highly satiating diet. However, the energy-restricted diet does not allow improvement of satiety efficiency in individuals who would benefit the most (i.e., the low satiety phenotype) as much as the satiating diet.

Interestingly, the results of the present study indicate that the satiety responsiveness influenced the changes in some eating behavior traits in response to an energy-restricted diet. Thus, in line with our hypothesis, individuals with LSR experienced less optimal changes in cognitive restraint and situational susceptibility to disinhibition (i.e., higher increase in cognitive restraint and some of its subscales and lower decrease in situational susceptibility to disinhibition). This is in contrast with our previous study [16] where changes in cognitive dietary restraint, disinhibition, and susceptibility to hunger were not influenced by satiety responsiveness in the context of a non-restrictive satiating diet (or control). Since a higher level of cognitive restraint has been associated with weight gain [41] and that disinhibition has been positively associated with poorer body-weight control [11], these results suggest that weight loss based on energy restriction may increase susceptibility to weight regain after the intervention in individuals with LSR. The higher increase in cognitive restraint in the LSR group may also suggest that the energy-restricted diet required more pronounced cognitive or psychological effort to be implemented or be followed by individuals having LSR. In the long-term, it could also be counterproductive for appetite control and even further decrease satiety responsiveness. It is difficult to compare these results with those of other studies, since to the best of our knowledge, no other study has specifically investigated eating and psychobehavioral profiles after weight loss based on energy restriction in LSR individuals. However, based on our previous results and the present study, these findings suggest that weight loss based on energy restriction in individuals with the low satiety phenotype triggers eating behaviors that may increase their risk of overeating, weight gain, or weight loss resistance. Therefore, weight loss interventions based on non-restrictive, highly satiating diets could be more optimal for these individuals. Recently, mindfulness interventions, as well as interventions including hunger training have also been shown to produce beneficial effects on body weight loss and eating behavior traits [42,43]. It is also important to note that similar changes were observed in both satiety responsiveness phenotypes for many eating behavior and psychobehavioral traits. For instance, both groups had a decrease in susceptibility to hunger as well as in food cravings as a trait. This is in line with what is generally observed in other weight loss studies based on energy restriction [20,44,45,46]. These results suggest that energy restriction could also have positive outcomes on eating behavior and psychobehavioral variables, yet no other study has looked at these responses in individuals with low satiety responsiveness. Thus, future work should be designed to specifically address altered satiety responsiveness during weight loss and maintenance.

Even though these studies were not primarily designed to specifically address satiety responsiveness, the secondary analysis of different studies which have used similar weight loss interventions (i.e., energy restriction of 500 to 700 kcal/day) represents a unique opportunity to address the impact of weight loss intervention on body weight loss, satiety efficiency, eating behavior traits, and psychobehavioral factors in a clinical weight loss population. The present findings are limited to a small sample of individuals who are overweight or obese, which limits the generalization of this study; however, it provides insight into potential targets for weight management. 

## 5. Conclusions

In conclusion, this study showed that differences in satiety efficiency exist in individuals who are overweight or obese and that the low satiety phenotype is associated with a few unfavorable eating and psychobehavioral traits. Although this profile does not appear to influence body weight loss in response to an energy-restricted diet, it may trigger undesirable changes in some eating behavior traits, which could increase their susceptibility to weight regain. 

## Figures and Tables

**Table 1:** Description of the three initial studies.

	Study 1 [22]	Study 2 [23]	Study 3 [21]
*n* ^1^	29	18	53
Women, *n* (%)	29 (100)	11 (61.1)	31 (58.5)
Control group intervention	Diet + placebo	Diet + placebo	Diet + placebo
Prescribed energy-restriction (diet), kcal/day	−700	−700	−500
Duration, weeks	15	15	12
Consultation with the dietitian	Each 2 weeks	Each 2 weeks	Each 2 weeks
Questionnaires ^2^			
TFEQ	x	x	x
BES			x
FCQ (S and T)			x
PSQI	x	x	x
BDI	x	x	x
STAI			x
Anthropometric measurements ^2^	x	x	x
Standardized breakfast and VAS measurements ^2^	x	x	x
Buffet-type meal ^2^	x	x	x

^1^ The present study only includes the control groups of the three initial studies. ^2^ These measures were taken before and after the weight loss intervention. TFEQ: Three-Factor Eating Questionnaire, BES: Binge Eating Scale, FCQ (S and T): State and Trait Food Craving Questionnaire, PSQI: Pittsburgh Sleep Quality Index, STAI: State-Trait Anxiety Inventory, VAS: 150 mm visual analogue scale measuring appetite sensations, x: measurement taken during the study.

**Table 2:** Baseline participant characteristics.

	All (*n* = 100)	LSR (*n* = 50)	HSR (*n* = 50)	*p* ^1^
Women, % (*n*)	71.0 (71)	54.0 (27)	88.0 (44)	0.0002
Men, % (*n*)	29.0 (29)	46.0 (23)	12.0 (6)	
Age, years	38.7 ± 8.7	37.8 ± 9.5	39.6 ± 7.8	0.30
**Anthropometric measures**				
Weight, kg	91.4 ± 14.4	95.6 ± 15.0	87.1 ± 12.5	0.15
Height, cm	165.7 ± 8.9	168.1 ± 9.8	163.3 ± 7.1	0.69
BMI, kg/m^2^	33.2 ± 3.6	33.7 ± 3.9	32.6 ± 3.3	0.11
Waist circumference, cm	103.4 ± 10.5	106.3 ± 11.2	100.5 ± 8.8	0.18
**Satiety Quotient (SQ)**				
SQ mean, mm/100 kcal	10.4 ± 5.4	6.0 ± 2.6	14.8 ± 3.5	<0.0001
SQ desire to eat, mm/100 kcal	10.7 ± 6.5	6.3 ± 4.7	15.1 ± 5.0	<0.0001
SQ hunger, mm/100 kcal	10.7 ± 6.5	5.6 ± 4.0	15.8 ± 4.0	<0.0001
SQ fullness, mm/100 kcal	12.0 ± 7.1	7.7 ± 6.0	16.3 ± 5.3	<0.0001
SQ PFC, mm/100 kcal	8.2 ± 5.4	4.3 ± 3.3	12.0 ± 4.3	<0.0001
**Fasting Appetite Sensations**				
Fasting desire to eat, mm	92.7 ± 38.8	78.4 ± 41.5	107.0 ± 30.0	<0.0001
Fasting hunger, mm	93.9 ± 38.1	76.5 ± 40.5	111.3 ± 25.8	<0.0001
Fasting fullness, mm ^2^	25.0 ± 28.4	32.6 ± 32.7	17.3 ± 20.9	0.049
Fasting PFC, mm	87.8 ± 32.0	80.3 ± 34.8	95.2 ± 27.3	0.0004

Values are presented as % (*n*) or means ± standard deviations ^1^ Differences between the LSR and HSR groups. ^2^ Analysis performed on log-transformed data. Except for age and sex, all statistical analyses were adjusted for sex. LSR: low satiety responsiveness, HSR: high satiety responsiveness, PFC: prospective food consumption.

**Table 3:** Eating behavior traits and psychobehavioral factors at baseline (pre) and after weight loss (post) in the LSR and HSR groups.

	LSR		HSR		*p* Group	*p* Time	*p* Group × Time
	Pre ^1^	Post ^2^	Pre	Post ^2^			
**Three-Factor Eating Questionnaire**							
***Cognitive restraint*** (0–21)	7.0 ± 3.4	12.3 ± 3.8 ****	8.8 ± 4.3	12.5 ± 4.0 ****	0.51	<0.0001	0.02
Flexible control (0–7)	1.8 ± 1.6	4.2 ± 1.9 ****	2.7 ± 1.6	4.3 ± 1.5 ****	0.44	<0.0001	0.02
Rigid control (0–7)	2.4 ± 1.5	3.4 ± 1.6	2.8 ± 1.7	3.6 ± 1.8	0.99	<0.0001	0.28
Strategic dieting behavior (0–4) ^3^	0.4 ± 0.7	1.9 ± 1.4 ****	0.9 ± 1.1	1.8 ± 1.2 ***	0.50	<0.0001	0.04
Attitude to self-regulation (0–5)	2.5 ± 1.2	3.5 ± 1.1	2.9 ± 1.5	3.4 ± 1.5	0.99	<0.0001	0.07
Avoidance of fattening foods (0–4)	2.0 ± 1.2	3.0 ± 1.0 ****	2.3 ± 1.1	2.7 ± 1.1	0.62	<0.0001	0.01
***Disinhibition*** (0–16)	8.5 ± 3.1	6.9 ± 2.8	8.5 ± 2.9	6.1 ± 2.8	0.25	<0.0001	0.20
Habitual susceptibility (0–5)	1.7 ± 1.3	1.0 ± 0.9	1.7 ± 1.5	1.0 ± 1.0	0.59	<0.0001	0.67
Emotional susceptibility (0–3)	1.7 ± 1.3	1.2 ± 1.3	1.9 ± 1.1	1.2 ± 1.2	0.30	<0.0001	0.42
Situational susceptibility (0–5)	3.3 ± 1.4	2.7 ± 1.4 **	3.3 ± 1.1	2.1 ± 1.4 ****	0.80	<0.0001	0.02
***Susceptibility to hunger*** (0–14)	6.7 ± 3.6	4.2 ± 3.2	5.2 ± 2.9	2.8 ± 2.2	0.04	<0.0001	0.84
Internal locus of hunger (0–6)	2.2 ± 1.7	1.4 ± 1.6	2.0 ± 1.6	0.8 ± 1.1	0.19	<0.0001	0.42
External locus of hunger (0–6)	3.3 ± 1.8 ^††^	1.9 ± 1.6	2.3 ± 1.5	1.2 ± 1.1	0.01	<0.0001	0.36
**Binge Eating Scale (BES)**							
Binge Eating (0–46)	12.7 ± 6.7	9.5 ± 6.3	10.3 ± 5.2	6.5 ± 5.0	0.08	<0.0001	0.52
**State-Trait Food Craving Questionnaire**							
***State Food Craving Questionnaire***							
Desire to eat (3–15)	7.2 ± 3.3	6.9 ± 3.1	6.3 ± 3.1	5.7 ± 2.5	0.74	0.20	0.49
Anticipation of Positive Reinforcement (3–15)	6.2 ± 2.7	6.1 ± 2.2	5.7 ± 2.7	4.9 ± 2.1	0.37	0.17	0.35
Anticipation of Relief from Negative states (3–15) ^3^	5.5 ± 2.3	5.4 ± 2.4	4.8 ± 2.5	4.5 ± 2.2	0.33	0.59	0.78
Lack of Control over Eating (3–15)	6.4 ± 3.0	5.7 ± 2.6	6.1 ± 2.6	5.0 ± 2.0	0.74	0.006	0.41
Craving as a Physiological State (3–15)	7.8 ± 3.1	6.7 ± 2.1	5.8 ± 2.5	5.5 ± 1.8	0.04	0.05	0.30
***Trait Food Craving Questionnaire***							
Intention and planning to consume food (3–18)	8.3 ± 2.9	7.5 ± 2.4	8.0 ± 1.6	7.0 ± 2.3	0.49	0.02	0.85
Anticipation of Positive Reinforcement (5–30)	15.6 ± 3.6	13.9 ± 4.5	13.8 ± 3.1	12.4 ± 2.7	0.11	0.02	0.91
Anticipation of Relief from Negative states (3–18)	7.8 ± 2.9	6.6 ± 2.4	6.7 ± 2.0	5.6 ± 1.8	0.05	0.003	0.91
Lack of Control over Eating (6–36)	17.0 ± 6.5	14.6 ± 5.6	15.0 ± 3.8	12.5 ± 2.9	0.11	<0.0001	0.75
Thoughts or Preoccupation with Food (7–42)	16.4 ± 5.4	15.2 ± 5.5	16.0 ± 5.5	14.0 ± 5.2	0.61	0.009	0.37
Craving as a Physiological State (4–24)	13.6 ± 3.8	12.7 ± 3.1	13.7 ± 2.2	12.0 ± 1.8	0.89	0.0002	0.12
Emotions that may be experienced (4–24)	11.2 ± 5.0	10.6 ± 4.7	10.6 ± 2.9	8.5 ± 2.7	0.07	0.006	0.07
Cues that may trigger food cravings (4–24)	15.5 ± 4.7	13.7 ± 4.7	14.3 ± 2.8	12.4 ± 2.4	0.33	<0.0001	0.66
Guilt that may be experienced (3–18)	8.8 ± 3.6	8.5 ± 3.1	8.3 ± 2.2	7.1 ± 2.7	0.09	0.06	0.26
**Pittsburg Sleep Quality Index (PSQI)**							
Sleep Quality (total score) (0–21) ^3^	5.1 ± 2.4 ^††^	4.0 ± 2.1	3.9 ± 2.5	3.3 ± 1.9	0.02	0.002	0.24
Sleep Duration (Hours)	7.2 ± 0.8 ^†^	7.4 ± 0.9	7.6 ± 0.8	7.5 ± 0.8	0.11	0.28	0.25
**Beck Depression Inventory (BDI)**							
Depressive symptoms (0–63) ^3, 4^	5.5 ± 6.2	4.3 ± 4.2	3.9 ± 4.0	3.9 ± 3.6	0.43	0.71	0.10
**State-Trait Anxiety Inventory**							
Present-state anxiety (20–80) ^4^	28.9 ± 6.3 ^†††^	29.7 ± 9.0	26.0 ± 3.4	25.1 ± 4.9	0.002	0.58	0.48
General-state anxiety (20–80)	38.8 ± 7.6	36.0 ± 7.4	35.2 ± 6.1	32.7 ± 4.6	0.04	0.002	0.93

LSR: Low satiety responsiveness, HSR: high satiety responsiveness. Values are unadjusted and presented as means ± standard deviations. Three-Factor Eating Questionnaire (cognitive restraint, disinhibition and susceptibility to hunger): Pre *n* = 90 to 98, Post *n* = 96 to 100; Binge Eating Scale: Pre *n* = 43, Post *n* = 46; State and Trait Food Craving questionnaire: Pre *n* = 49 to 52, Post 50 to 52; Pittsburg Sleep Quality Index (PSQI), Total score: Pre *n* = 93, Post *n* = 94; Sleep duration: Pre *n* = 98, Post *n* = 96; Depressive symptoms: Pre *n* = 92; Post *n* = 93; Present State Anxiety: Pre *n* = 51, Post *n* = 49; General State Anxiety: Pre *n* = 48, Post *n* = 52. *p* values for group, time and group **×** time interaction are adjusted for sex, baseline BMI in all models and for weight loss duration and prescribed energy restriction (i.e., condition A; −700 kcal/day of 15 weeks; condition B: −500 kcal/day for 12 weeks) in models related to the TFEQ, BDI and PSQI since the other questionnaires were only available in one of the initial studies. ^1^ Significant difference vs. baseline HSR using GLM adjusted for sex and baseline BMI, ^†^ *p* = 0.05; ^††^ *p* < 0.05, ^†††^ *p* < 0.01. ^2^ Significant difference vs. baseline as per Tukey-Kramer post hoc test from the Mixed model for repeated measures, * *p* < 0.05, ** *p* < 0.01, *** *p* < 0.001, **** *p* < 0.0001. ^3^ Analysis for group difference in baseline values performed on log-transformed data (GLM). ^4^ Analysis performed on log-transformed data (Mixed model for repeated measures).
